# Research overview and hotspots in the field of recurrent miscarriage: A literature-mining study

**DOI:** 10.3389/fmed.2025.1605088

**Published:** 2025-10-09

**Authors:** Chaoyan Shen, Juan Zhang, Miaomiao Zhang, Quan Yuan, Yan Lu, Lele Miao, Wei Wang

**Affiliations:** ^1^Department of Ultrasound, Jining No.1 People’s Hospital, Jining, Shandong; ^2^Department of Hematology, Jining No.1 People’s Hospital, Jining, Shandong, China; ^3^Department of Pathology, Jining No.1 People’s Hospital, Jining, Shandong, China; ^4^Department of Thyroid and Breast Surgery, Jining No.1 People’s Hospital, Jining, Shandong, China; ^5^Department of Clinical Laboratory Medicine, Jining No.1 People’s Hospital, Jining, Shandong, China

**Keywords:** recurrent miscarriage, research overview, research hotspots, bibliometric, literature mining

## Abstract

Utilizing bibliometric analysis, this study analyzed relevant literature in the field of recurrent miscarriage and systematically reviewed the research overview and hotspots in this field from 2015 to 2024. A total of 3,114 articles were retrieved from the Web of Science Core Collection (WoSCC) database. The analysis demonstrated that the number of papers published in this field was on the rise. Notably, China, Shanghai Jiao Tong University, the American Journal of Reproductive Immunology, and researcher Jing Yang were identified as the most prolific contributors. The most cited researchers and journals were Siobhan Quenby and Fertility and Sterility respectively. The keyword cluster analysis revealed four primary research directions: the pathophysiological processes and associated molecular mechanisms of recurrent miscarriage, the influence of antiphospholipid syndrome and coagulation dysfunction, the role of chromosomal abnormalities and assisted reproductive technologies, and investigations into immunological mechanisms. Co-citation analysis identified key research hotspots in recurrent miscarriage, encompassing risk assessment, pathogenesis, advanced diagnostic methods, biomarker discovery, and single-cell sequencing applications. This study provides a multi-dimensional perspective and comprehensive summary of the research progress in the field of recurrent miscarriage, highlights the importance of multidisciplinary cooperation in this area, and serves as a valuable reference for researchers.

## Introduction

In the field of reproductive medicine, recurrent miscarriage poses a significant and persistent clinical challenge. As a complication of pregnancy that significantly impacts women’s reproductive and mental health, the global incidence of recurrent miscarriage is estimated to be between 1 and 5% (1). Recurrent miscarriage imposes substantial psychological and financial burdens on patients and their families, in addition to its profound societal impact. Consequently, research in this area represents a focus within reproductive medicine. The etiology of recurrent miscarriage is multifaceted, encompassing genetic factors (2), immune abnormalities (3), endocrine disorders (4), abnormal anatomical structure (5), abnormal coagulation function (6) and infection (7). Nevertheless, approximately 50% of cases remain idiopathic (8). Comprehensive investigation into the pathogenesis of recurrent miscarriage is essential for enhancing the precision of etiological diagnosis and providing a scientific foundation for clinical interventions.

In recent years, some advancements have been made in the diagnosis and treatment of recurrent miscarriage. Regarding diagnostic methodologies, genetic factors are recognized as significant contributors to recurrent miscarriage. Research indicates that chromosomal abnormalities are among the primary causes of miscarriage, particularly in early pregnancy (9). Chromosomal microarray analysis (CMA) and whole exome sequencing (WES) have enhanced the detection of genetic variations associated with miscarriage, thereby offering patients more precise diagnoses and genetic counseling (9, 10). In terms of treatment strategies, advancements in immunology have introduced novel perspectives for addressing recurrent miscarriage. There is an established correlation between maternal-fetal immunology and unexplained recurrent miscarriage, which holds promise for the development of personalized treatment strategies (11). Although effective diagnostic tools for immunological etiologies are currently lacking and the efficacy and safety of lymphocyte-based immunotherapy and intravenous immunoglobulin (IVIG) remain controversial, these studies provide a critical foundation for developing future treatment strategies (12).

The study of recurrent miscarriage holds not only great scientific significance but also far-reaching social significance, as it improves patients’ reproductive health and quality of life. Firstly, the high prevalence and complexity of recurrent miscarriage present an urgent challenge within the domain of reproductive medicine. A comprehensive understanding of the pathogenesis of recurrent miscarriage is crucial for improving the precision of etiological diagnosis and establishing a scientific foundation for effective clinical interventions. Secondly, research on recurrent miscarriage necessitates multidisciplinary collaboration, thereby advancing the fields of reproductive medicine, immunology, genetics, and microbiology. Such collaboration enables researchers to gain a more comprehensive understanding of the pathogenesis of recurrent miscarriage and to develop more effective diagnostic and therapeutic strategies. Lastly, the study of recurrent miscarriage is intricately linked to contemporary social and technological advancements. With the emergence of precision medicine, formulating personalized treatment plans based on individual etiology and risk stratification has become a pivotal direction in the management of recurrent miscarriage. Precision medicine approaches can enhance therapeutic efficacy while minimizing unnecessary medical interventions and associated side effects. This study employs bibliometric analysis to examine recurrent miscarriage research from the past decade, aiming to identify research trends and hotspots to inform future advancements in the field.

## Data and bibliometrics software

All data were obtained from the Web of Science Core Collection (WoSCC) database. The literature search for this study was executed utilizing the following query: TS = (“recurrent abortion” OR “habitual abortion” OR “recurrent miscarriage” OR “recurrent early pregnancy loss” OR “recurrent spontaneous abortion” OR “recurrent pregnancy loss”). The inclusion criteria were defined as follows: (a) publication date range: January 1, 2015, to December 31, 2024; (b) language of publication: English; and (c) type of document: article. The exclusion criteria encompassed: (a) literature not related to the subject of this study; and (b) publication types other than articles. The processes of literature retrieval and screening were conducted independently by two investigators, with any discrepancies resolved through consultation with a third researcher. Following the removal of duplicates using bibliometric software, a total of 3,114 articles were included in the final analysis. A comprehensive summary of the retrieval formula and process is provided in Supplementary Table 1. The bibliometric and statistical software used in this study include CiteSpace, VOSviewer, and Microsoft Office Excel 2019.

## Bibliometrics and visual analysis in the recurrent miscarriage research field

**FIGURE 1:**
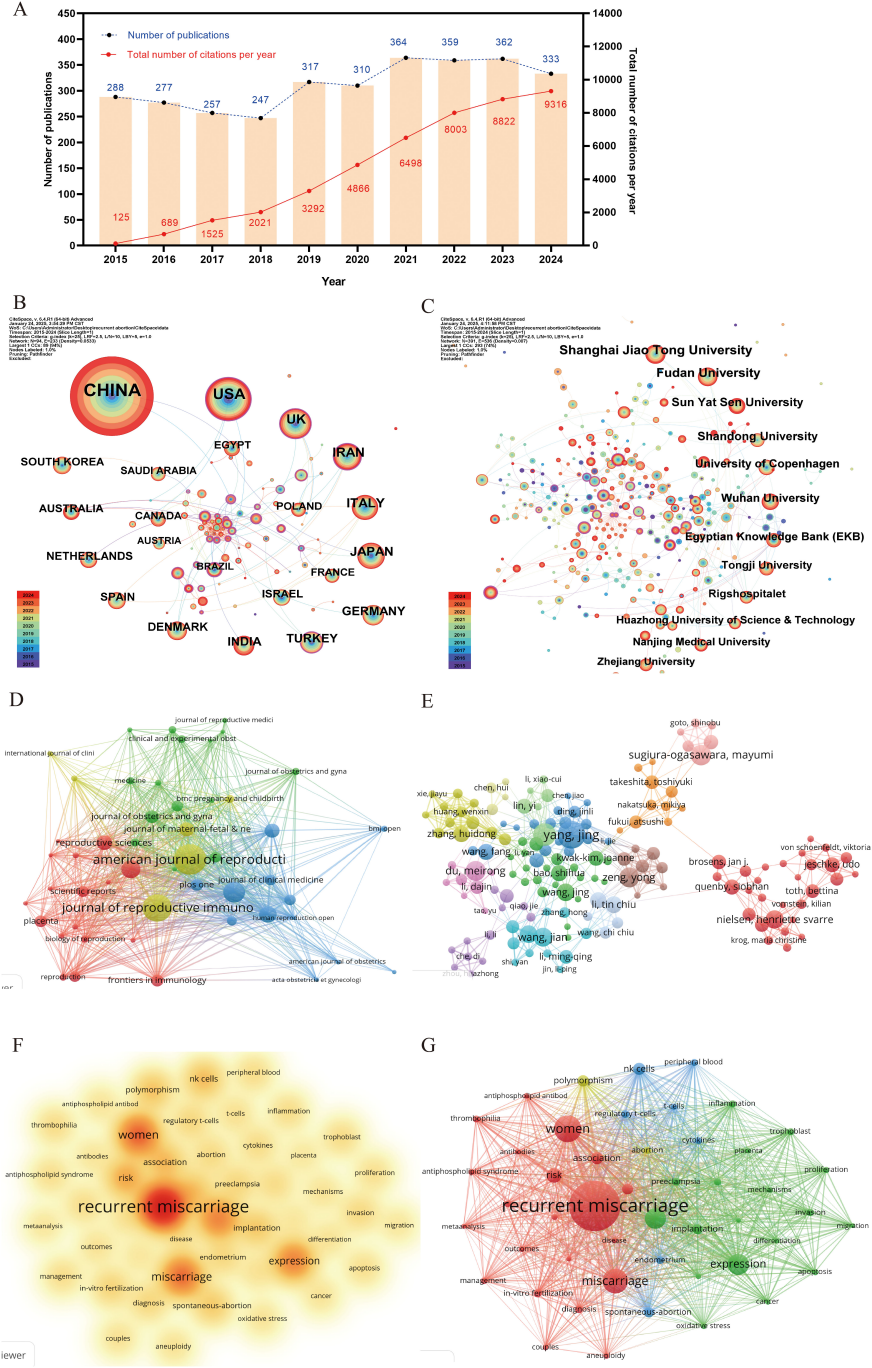
The bibliometrics analysis of recurrent miscarriage. (**A**) The trend of annual publication number and citation frequency of articles in this research field. It shows the number of publications and cited times every year. The co-occurrence map of countries **(B)**, institutions **(C)** and journals **(D)** about the research field. The visualization map of authors **(E)** about the research field. The co-occurrence density map **(F)** of keywords. **(G)** The network visualization of keywords. Each node in the graph represents a separate individual, such as country, institution, author, and keyword, and the size of the node represents the frequency of occurrence. Connecting lines indicate co-occurrence relationships between different individuals.

**FIGURE 2:**
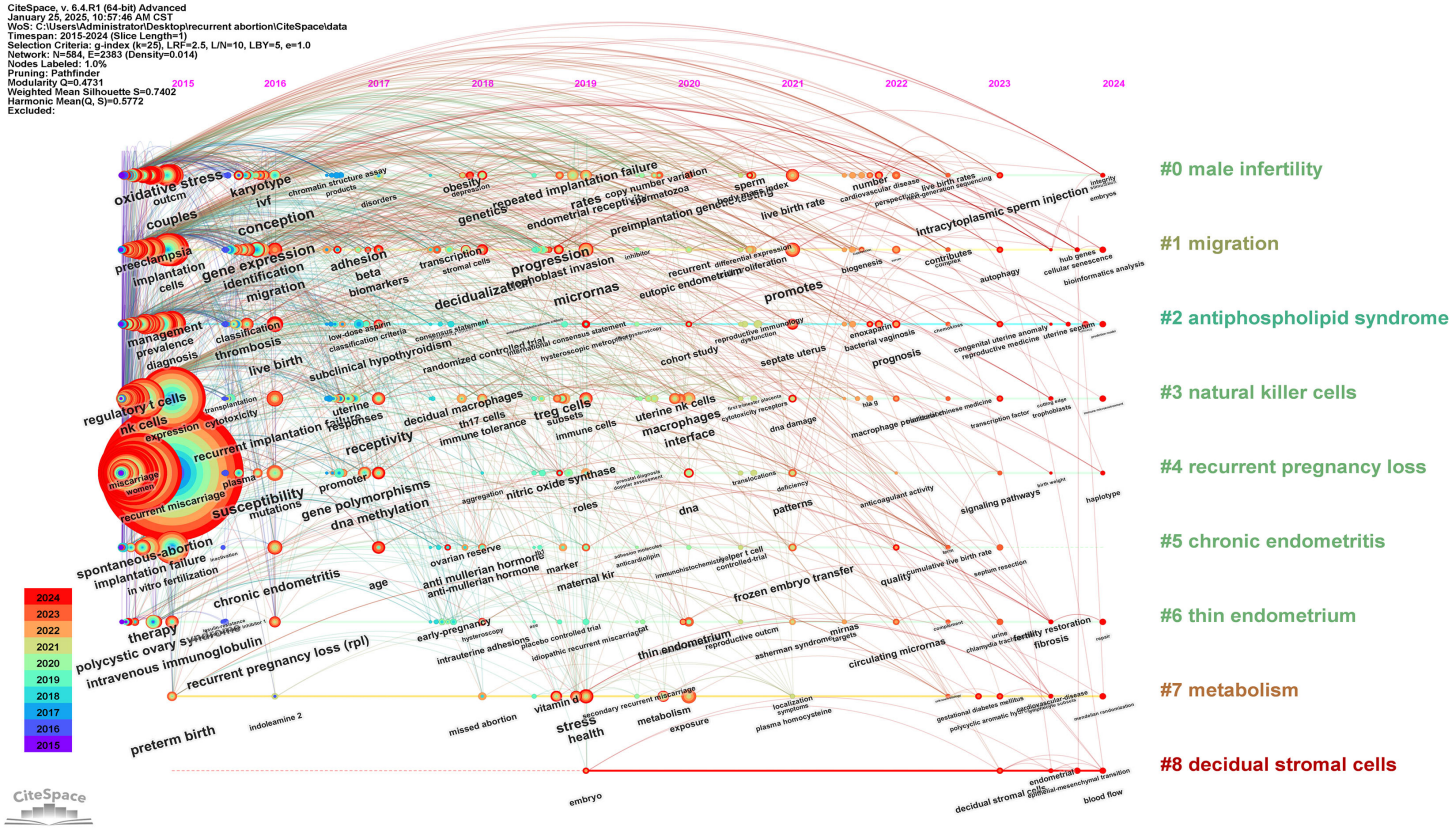
The timeline viewer of keywords. This graph shows the evolution of keywords in the research field of recurrent abortion from 2015 to 2024. Each node in the diagram represents a keyword, and the size of the node indicates the frequency of the keyword in a specific year, that is, the research popularity. Connecting lines indicate the co-occurrence relationship between keywords, which reflects the frequency of researchers discussing these concepts at the same time in papers. Through this picture, we can intuitively observe the hotspots transfer and the latest keywords in this field.

Over the past decade, a total of 3,114 articles were published in the field of recurrent miscarriage, accumulating 45,157 citations and achieving an h-index of 74. The trend analysis of annual publication volume indicated a progressive increase in research output within this domain (Figure 1A), suggesting a gradual rise in scholarly interest. The countries, institutions, journals and authors that published the most articles were China (*n* = 1,237), Shanghai Jiao Tong University (*n* = 111), American Journal of Reproductive Immunology (*n* = 143) and Jing Yang (*n* = 35) (Figures 1B–E and Supplementary Table 2). Fertility and Sterility emerged as the most frequently cited journal, with 3,718 citations (Supplementary Table 3). Siobhan Quenby was identified as the most cited author, with 1,352 citations (Supplementary Table 4). The keyword “recurrent miscarriage” occurred with the highest frequency (*n* = 1,947), followed by “women” (*n* = 791), “vacuum” (*n* = 625), and “miscarriage” (*n* = 709) (Figure 1F and Supplementary Table 5). Through keyword cluster analysis, four distinct clusters were identified (Figure 1G), each representing a primary research direction. These included: (1) investigations into the pathophysiological processes and molecular mechanisms underlying recurrent miscarriage (red cluster); (2) exploration of the role of antiphospholipid syndrome (APS) and associated coagulation dysfunction in recurrent miscarriage (green cluster); (3) examination of the relationship between recurrent miscarriage and chromosomal abnormalities, alongside the application of assisted reproductive technology (blue cluster); and (4) studies on the immunological mechanisms of recurrent miscarriage (yellow cluster). The construction of a timeline diagram of keywords elucidates the temporal distribution and evolutionary trajectory of these keywords, as well as significant topics. For instance, Figure 2 effectively illustrates the progression of keywords and research themes that emerged each year.

## Research hotspots in the field of recurrent miscarriage from 2015 to 2024

To further investigate the prominent research areas within this field, we performed a co-citation analysis of the relevant literature (Supplementary Table 6) (13–22), which led to the identification of six potential major research hotspots.

### The risk assessment of recurrent miscarriage

It mainly includes maternal factors and immune factors. Magnus et al. (13) reported a significantly increased risk of miscarriage among women over 30 years of age, with adverse pregnancy outcomes such as previous preterm birth, stillbirth, cesarean section, and gestational diabetes further elevating the risk of miscarriage. It is suggested that advanced maternal age and a history of adverse pregnancy outcomes constitute critical risk factors for recurrent miscarriage. Regarding immunological factors, research primarily focuses on the roles of immune cells and their associated molecules at the maternal-fetal interface in recurrent miscarriage. This includes alterations in the proportions of immune cell subsets (e.g., regulatory T cells and NK cells) in peripheral blood and decidual tissue, as well as variations in the expression of cytokines (e.g., interleukin-6, IL-10, and TNF-α) and immunomodulatory molecules (e.g., CTLA-4 and TGF-β1). These immunological changes influence maternal and fetal immune tolerance and are closely associated with recurrent miscarriage (16, 17, 22). The role of immunological factors in recurrent miscarriage has also garnered significant attention. Maternal-fetal immune imbalance may lead to embryo rejection and reproductive failure. Immunoregulation and immunosuppressive therapies have demonstrated promising potential in enhancing pregnancy outcomes and increasing live birth rates (11). Consequently, the improvement of diagnosis and treatment strategies for immune factors may provide a new direction for the management of recurrent miscarriage.

### The pathogenesis of recurrent miscarriage

(a) The mechanism of cell biology. Exploring the changes of trophoblast migration, invasion and epithelial-mesenchymal transition (EMT) in recurrent miscarriage. For instance, miR-27a-3p modulates trophoblast migration and invasion by downregulating the expression of ubiquitin-specific protease 25 (USP25), thereby contributing to the pathogenesis of recurrent miscarriage (19).

(b) The immune regulatory mechanism. The cellular and molecular characterization of decidua and peripheral blood leukocytes at the single-cell level revealed abnormal distribution and functional alterations of immune cell subsets in patients with recurrent miscarriage, as well as imbalances in cell-to-cell communication and immune regulatory networks (16, 17). For example, alterations in the proportion and functionality of decidual natural killer cell (dNK) subsets, coupled with dysregulated ligand-receptor interactions between immune cells, can lead to abnormal immune activation or a loss of immune tolerance at the maternal-fetal interface, ultimately resulting in pregnancy loss (16, 17).

(c) Abnormal changes of endometrium and decidua. For instance, the deficiency of endometrial stem cells and the accelerated senescence of stromal cells may constrain endometrial differentiation capabilities, thereby impacting pregnancy outcomes (18); during the implantation window, aberrant phenomena such as stress responses, cellular aging, and progesterone resistance in decidual cells compromise the maternal-fetal interface, potentially resulting in miscarriage (20). Furthermore, further investigation is necessary to elucidate the aberrant response of endometrial cells to decidualization signals and the association between altered methylation status of pertinent genes and recurrent miscarriage (18).

(d) The metabolic mechanism. Metabolites also play a crucial role in recurrent spontaneous abortion (RSA). For instance, succinic acid concentration in chorionic villi is correlated with RSA risk. Aberrant levels of this metabolite can impair extravillous trophoblast function, thereby increasing the likelihood of pregnancy loss (21).

The precise etiology of recurrent miscarriage remains incompletely understood. In addition to previously discussed mechanisms, other potential contributing factors to recurrent miscarriage include genetic abnormalities, endocrine dysfunctions, anatomical anomalies, coagulation disorders, and infections. Genetic factors, in particular, are pivotal in recurrent miscarriage. Research indicates that that chromosome abnormality is one of the main causes of abortion, especially when parents or fetuses have chromosome defects in structure or quantity (2). Furthermore, gene mutations and polymorphisms may be associated with recurrent miscarriage. The identification of these genetic markers can facilitate improved risk prediction and etiological diagnosis (23). Endocrine disorders represent another significant contributor to recurrent miscarriage. Disruptions within the endocrine system can alter reproductive hormone secretion, adversely affecting pregnancy maintenance and progression (4). For instance, endocrine conditions such as polycystic ovary syndrome (PCOS) are frequently linked to an elevated risk of miscarriage (4). Abnormal anatomical structure, such as uterine malformation, is also considered as one of the potential causes of recurrent miscarriage (5). Abnormal anatomical structure may affect the normal implantation and development of embryos. Abnormal coagulation function, particularly thrombophilia, is closely associated with recurrent miscarriage. A propensity for thrombosis can result in inadequate placental blood flow, thereby impacting normal fetal development (6). Additionally, infection constitutes a significant factor in recurrent miscarriage, as it may induce abortion either by directly affecting the placenta or by eliciting a maternal immune response (7). The etiology of recurrent miscarriage is complex and multifaceted, necessitating multidisciplinary diagnostic and therapeutic approaches to enhance pregnancy success rates.

### The improvement of diagnostic methods for recurrent miscarriage

Popescu et al. (15) conducted a single-center prospective cohort study to evaluate the efficacy and cost-effectiveness of integrating microarray analysis of chromosome 24 from aborted tissues with the standard evaluation method recommended by the American Society for Reproductive Medicine (ASRM) in diagnosing recurrent miscarriage. The findings indicate that the combined approach enables approximately 95% of patients to ascertain the cause of miscarriage and demonstrates greater cost-effectiveness when the 24-chromosome microarray analysis is performed initially. This combined method offers a more effective strategy for diagnosing recurrent miscarriage. Furthermore, Li et al. (24) developed a nomogram model designed to predict the risk of subsequent pregnancy loss in patients with recurrent miscarriage. By integrating nine independent risk predictors, the model can accurately assess the risk of pregnancy loss of patients. The diagnosis of recurrent miscarriage needs to consider many factors. A comprehensive evaluation of these factors enables a more accurate determination of the etiology behind pregnancy loss and facilitates the development of personalized treatment plans for patients (25).

### The exploration of biomarkers related to recurrent miscarriage

In recent years, biotechnological advancements have significantly accelerated the discovery of biomarkers associated with recurrent miscarriage. Certain biomarkers are anticipated to serve as tools for the early diagnosis and monitoring of recurrent miscarriage. These biomarkers encompass specific genes (e.g., HMGB2), microRNAs (e.g., miR-27a-3p), alterations in the proportions of immune cell subsets (e.g., the Th17/Treg cell ratio), and metabolite levels (e.g., succinic acid) (16, 17, 19, 21). Furthermore, biomarkers related to oxidative stress and ferroptosis are also considered to play a significant role in the pathophysiology of recurrent miscarriage. Through the analysis of transcriptome data from patients experiencing recurrent miscarriage, researchers have identified several differentially expressed genes (DEGs) associated with ferroptosis and oxidative stress, including PTPN6, GJA1, CPT1A, and CREB3L1. The identification of these genes may provide a critical theoretical foundation for improving the diagnosis and treatment of recurrent miscarriage (26). The role of long-chain non-coding RNA (lncRNA) in recurrent miscarriage is becoming increasingly elucidated. Notably, the polymorphism rs13252298 in the PRNCR1 gene has been associated with a reduced susceptibility to recurrent miscarriage, suggesting its potential as a biomarker for assessing miscarriage risk (27). The investigation of biomarkers related to recurrent miscarriage provides novel insights into its pathological mechanisms and presents potential targets for future diagnostic and therapeutic strategies. Nonetheless, further research is essential to validate the clinical efficacy and reliability of these markers.

### The application of single-cell sequencing technology in the study of recurrent miscarriage

Recurrent miscarriage is a clinically heterogeneous disorder with a complex pathogenesis. The application of single-cell sequencing technology enables an in-depth analysis of cellular heterogeneity within tissues associated with miscarriage, facilitating the identification of specific cell subsets and gene expression profiles pertinent to recurrent miscarriage. This approach offers a novel perspective for elucidating the pathological mechanisms underlying recurrent miscarriage and may uncover potential diagnostic biomarkers and therapeutic targets. Firstly, single-cell RNA sequencing technology can be employed to characterize the heterogeneity of immune cells, particularly macrophages, in miscarriage tissues. Research indicates that macrophages play a crucial role in these tissues, and alterations in their gene expression patterns may be intricately linked to the incidence of miscarriage (28). Single-cell sequencing facilitates the identification of specific macrophage marker genes. The differential expression profiles of these genes can subsequently be compared between patients with recurrent miscarriage and healthy controls with normal pregnancy outcomes. This provides a new possibility for the diagnosis and treatment of abortion. Secondly, single-cell sequencing technology serves as a powerful tool for investigating alterations in various cell types within abortion tissues. For instance, the technology can reveal the morphological and transcriptional changes of trophoblast cells during implantation (29). This analysis enhances our understanding of the mechanisms underlying embryo implantation failure and offers valuable insights for studying early pregnancy pathology. Moreover, the application of single-cell sequencing in abortion research extends to the identification and functional validation of genes associated with abortion. By integrating whole-exome sequencing with single-cell sequencing, researchers can identify candidate genes linked to abortion and verify their roles through functional experiments (30). This approach not only aids in uncovering the genetic causes of abortion but also lays the groundwork for developing personalized diagnostic and therapeutic strategies.

The application of single-cell sequencing technology in the investigation of recurrent miscarriage offers a novel perspective for elucidating the pathological mechanisms underlying abortion. This approach has the potential to advance the diagnosis and treatment of recurrent miscarriage. By conducting a comprehensive analysis of cellular heterogeneity and gene expression profiles within abortion tissues, researchers can gain a deeper understanding of the complexity associated with recurrent miscarriage, thereby establishing a foundation for future clinical applications.

### The treatment of recurrent miscarriage

In recent years, significant advancements have been made in the diagnosis and treatment of recurrent miscarriage. The application of new methods such as immunotherapy, anticoagulation therapy, and assisted reproductive technology has provided more treatment options for patients. Firstly, the application of immunotherapy in addressing recurrent miscarriage has garnered increasing attention. The modulation of the immune system is crucial for successful embryo implantation and the maintenance of pregnancy. Immunotherapy has the potential to improve pregnancy outcomes by modulating the maternal immune response, facilitating embryo acceptance, and mitigating immune-mediated attacks (11). Early clinical trials and meta-analyses have indicated that this therapy may enhance pregnancy outcomes (31–33). Consequently, paternal lymphocyte immunotherapy (LIT) was historically employed as a treatment for recurrent miscarriage, representing one of the initial therapeutic approaches. However, the efficacy of LIT has remained contentious due to the methodological limitations of earlier studies and the absence of robust randomized controlled trial (RCT) data. In 2018, drawing on additional clinical data and a meta-analysis conducted by Wong et al. (34), “ESHRE Guideline: Recurrent Pregnancy Loss (1)” advised against the use of lymphocyte immunotherapy for the treatment of unexplained recurrent miscarriage, citing its lack of significant efficacy and potential for serious adverse reactions.

In recent years, there has been significant interest in the use of corticosteroids and intravenous immunoglobulin (IVIG) for the treatment of recurrent miscarriage. Firstly, the updated ESHRE Guideline in 2022 pointed out that the repeated administration of high-dose IVIG during early pregnancy may enhance the live birth rate in women experiencing four or more unexplained recurrent miscarriages (35). One recent meta-analysis also demonstrated that, in comparison to the placebo group, the IVIG treatment group exhibited a significantly increased live birth rate (OR = 2.24, 95% CI = 1.68∼2.98, *p* < 0.00001) and a significantly reduced miscarriage rate (OR = 0.46, 95% CI = 0.22∼0.95, *p* = 0.04) (36). Furthermore, IVIG has been shown to modulate the balance of Th17/Treg cells, which may represent one of its mechanisms for improving pregnancy outcomes (37). Nevertheless, the use of IVIG is associated with a high incidence of adverse reactions, necessitating large-scale, multicenter randomized controlled trials to confirm its safety and efficacy (36). Secondly, the utilization of corticosteroids in the management of recurrent miscarriage demonstrates potential efficacy. A systematic review and meta-analysis indicated that oral corticosteroids could significantly enhance the rate of sustained pregnancies beyond 12 weeks gestation (log OR = 1.49, *p* = 0.01) and improve live birth rates (log OR = 0.9, *p* = 0.03) (38). Nevertheless, the limited number of studies constrains the robustness of these findings. Furthermore, the risk/benefit assessment of using glucocorticoids during the early pregnancy period remains unclear and warrants further in-depth investigation (38).

Anticoagulant therapy is also recognized as an effective intervention for recurrent miscarriage. This treatment can enhance endometrial blood supply and reduces thrombosis, thereby increasing the likelihood of successful embryo implantation and pregnancy maintenance (39). Notably, for patients diagnosed with antiphospholipid antibody syndrome (APS), the concurrent administration of low molecular weight heparin (LMWH) and aspirin is widely advocated, as it has been shown to significantly improve pregnancy outcomes (40). However, the application of LMWH in women with a history of recurrent miscarriage and hereditary thrombosis has always been a controversial topic. The ALIFE2 trial represents the sole multicenter, open-label, randomized controlled study conducted to date that targets this specific population. This trial investigated the efficacy of LMWH in treating women with recurrent miscarriage and hereditary thrombophilia (41). The findings indicated that LMWH did not significantly enhance the live birth rate among these women, and there was no notable difference in the incidence of adverse events between the treatment and control groups. Consequently, the researchers concluded that LMWH is not recommended for this particular population, nor is it advisable to screen women with a history of recurrent miscarriage for hereditary thrombophilia. This study holds substantial significance for informing clinical practice, as it aids in preventing unnecessary treatments and the associated waste of resources.

Assisted reproductive technology (ART) plays a significant role in managing recurrent miscarriage. ART encompasses various techniques such as *in vitro* fertilization (IVF) and embryo transfer, which facilitate conception for couples experiencing fertility challenges. Research indicates that the integration of ART with adjunctive therapies, such as immunotherapy, can improve the likelihood of successful pregnancies (42). Advancements in ART have enabled the implementation of selective single embryo transfer, thereby mitigating the risk of multiple gestations and enhancing pregnancy safety (43). Additionally, emerging treatment strategies, including stem cell therapy, show considerable promise for managing recurrent miscarriage. Stem cells possess the unique capabilities of self-renewal and multi-differentiation, which may contribute to improved pregnancy outcomes by repairing damaged endometrial tissue or modulating immune responses (44). Mesenchymal stem cells (MSCs), in particular, are regarded as having considerable potential for application in the treatment of recurrent miscarriage due to their immunomodulatory and immunosuppressive properties (45).

The management of recurrent miscarriage remains one of the most formidable challenges in reproductive medicine. Despite some advancements in treatment strategies, it is crucial to acknowledge that the evidence supporting most current therapeutic approaches is still limited. Among the 3,114 documents included in this study, those clearly labeled as “randomized controlled trial” or “clinical trial” accounted for only approximately 4%. Furthermore, these studies often suffer from limitations such as small sample sizes and single-center designs. High-quality randomized controlled trials (RCTs) with multi-center participation and large sample sizes are exceedingly rare. This paucity of robust RCT research undermines the ability to evaluate the efficacy and safety of many therapies with high-level evidence-based medicine, thereby impeding the development of clinical guidelines. Consequently, clinicians have to weigh between “possible effectiveness” and “unknown risk”. Future research should prioritize and actively advance the design and implementation of high-quality RCTs. These trials represent the gold standard for translating basic research findings and validating clinical hypotheses, serving as the foundation for developing authoritative clinical guidelines and optimizing patient management strategies. It is only through meticulously designed and effectively executed RCTs that we can overcome the existing treatment challenges and elevate the clinical management of recurrent miscarriage to a more precise, effective, and standardized level.

## Conclusion

A recent bibliometric analysis conducted by Jiang et al. (46) focused on the study of unexplained recurrent spontaneous abortion (URSA) from 2014 to 2024. While their study offers a valuable overview within the specific domain of URSA, our research encompasses a broader spectrum of recurrent miscarriage, addressing both explained and unexplained cases. This expanded scope allows for a more comprehensive analysis of research trends, international collaborations, and research hotspots, such as single-cell sequencing and novel biomarkers. In contrast to the study by Jiang et al., our research examines a larger dataset (3,114 publications vs. 586), providing a macroscopic perspective on the evolution of recurrent miscarriage research and complementing the critical insights on URSA offered by Jiang et al. Collectively, these studies offer complementary value: Jiang et al.’s work delves into the depth of specific diagnostic subgroups, whereas our research provides a broader overview of the entire field. Consequently, this combined approach furnishes a more holistic reference for understanding the research advancements and future directions in recurrent miscarriage over the past decade.

Recurrent miscarriage remains a significant challenge in reproductive medicine. Substantial research progress has been made in recent years across multiple aspects, offering new hope for improving clinical diagnosis, treatment, and ultimately, patient prognosis. In the investigation of etiology, research has progressed from examining traditional factors such as maternal age and pregnancy history to delving into cellular, molecular, and genetic levels. Within the realm of immunology, the dynamic alterations in immune cell subsets at the maternal-fetal interface and the imbalance of the immune regulatory network have emerged as significant areas of study. These investigations highlight the crucial role of impaired immune tolerance in recurrent miscarriage. In cell biology, the dysfunction of trophoblasts, abnormal differentiation and senescence of endometrial and decidual cells, and the influence of metabolites on cellular function offer new insights into pathogenesis. Diagnostic technologies are also advancing, with the integration of microarray analysis of 24 chromosomes in abortion tissue with traditional evaluation methods significantly enhancing the accuracy and efficiency of etiological diagnosis, thereby laying the groundwork for precise treatment. Concurrently, the identification of numerous potential biomarkers offers promising avenues for early warning and disease monitoring.

Despite significant advancements, research on recurrent miscarriage continues to face considerable challenges. Future investigations must delve deeper into the multifactorial etiological mechanisms underlying recurrent miscarriage and focus on the development of more precise diagnostic markers and effective therapeutic strategies. The integration of single-cell sequencing technology is anticipated to further advance this field, offering enhanced insights into the pathogenesis of recurrent miscarriage. Moreover, a multidisciplinary approach is essential, incorporating emerging technologies such as single-cell sequencing and gene editing to thoroughly analyze the underlying mechanisms. There is a pressing need to strengthen large-scale clinical research to validate and refine diagnostic methods and therapeutic strategies. Concurrently, innovative treatment strategies should be formulated based on the elucidated pathogenesis, aiming for a comprehensive breakthrough from etiological diagnosis to precise treatment. This approach has the potential to significantly reduce the incidence of recurrent miscarriage and assist more patients in achieving their reproductive aspirations.
