# Supplementary material for: Research overview and hotspots in the field of recurrent miscarriage: A literature-mining study
Source: Front Med (Lausanne). 2025 Oct 9;12:1605088. doi: 10.3389/fmed.2025.1605088 (PMC12546178; doi:10.3389/fmed.2025.1605088)
Supplement: Supplementary file 1 [file Table_1.docx]

**Table S1. Retrieval Formula**

| **Search Process** | **Count** |
| --- | --- |
| #1: TS = (“recurrent abortion*”) | 666 |
| #2: TS = (“habitual abortion*”) | 233 |
| #3: TS = (“recurrent miscarriage*”) | 3,613 |
| #4: TS = (“recurrent early pregnancy loss”) | 111 |
| #5: TS = (“recurrent spontaneous abortion*”) | 1,880 |
| #6: TS = (“recurrent pregnancy loss”) | 3,516 |
| #7: #6 OR #5 OR #4 OR #3 OR #2 OR #1 | 8,843 |
| Time: 2015-01-01 to 2024-12-31 | 4,726 |
| The language limit is English | 4,693 |
| The type is limited to article | 3,114 |
| Finally, a total of **3,114** articles were obtained | |

**Table S2. The top 10 countries and institutions in this research field**

| **Rank** | **country** | **Counts** | **Centrality** | **Institution** | **Counts** | **Centrality** |
| --- | --- | --- | --- | --- | --- | --- |
| 1 | CHINA | 1237 | 0.04 | Shanghai Jiao Tong University | 111 | 0.02 |
| 2 | USA | 378 | 0.12 | Fudan University | 95 | 0.02 |
| 3 | UK | 185 | 0.12 | Sun Yat Sen University | 68 | 0 |
| 4 | IRAN | 154 | 0.18 | Shandong University | 66 | 0 |
| 5 | ITALY | 150 | 0.08 | University of Copenhagen | 64 | 0 |
| 6 | JAPAN | 144 | 0.04 | Wuhan University | 64 | 0.02 |
| 7 | TURKEY | 102 | 0.11 | Egyptian Knowledge Bank (EKB) | 54 | 0.06 |
| 8 | INDIA | 102 | 0 | Tongji University | 49 | 0.04 |
| 9 | GERMANY | 102 | 0 | Rigshospitalet | 48 | 0.02 |
| 10 | DENMARK | 87 | 0.04 | Huazhong University of Science & Technology | 46 | 0.04 |

**Table S3. The top 10 journals in this research field**

| **Rank** | **Journal** | **Citations** | **Documents** |
| --- | --- | --- | --- |
| 1 | Fertility and Sterility | 3718 | 87 |
| 2 | Human Reproduction | 2614 | 67 |
| 3 | American Journal of Reproductive Immunology | 1869 | 143 |
| 4 | Journal of Reproductive Immunology | 1451 | 126 |
| 5 | Reproductive Biomedicine Online | 871 | 54 |
| 6 | Human Reproduction Open | 828 | 13 |
| 7 | Journal of Assisted Reproduction and Genetics | 805 | 73 |
| 8 | Plos One | 769 | 43 |
| 9 | Scientific Reports | 645 | 40 |
| 10 | Journal of Maternal-fetal & Neonatal Medicine | 641 | 54 |

**Table S4. The top 10 authors in this research field**

| **Rank** | **Cited author** | **Citations** | **Documents** |
| --- | --- | --- | --- |
| 1 | Siobhan Quenby | 1352 | 18 |
| 2 | Jan J. Brosens | 1118 | 17 |
| 3 | Saskia Middeldorp | 957 | 8 |
| 4 | Ole Bjarne Christiansen | 917 | 15 |
| 5 | Mariette Goddijn | 875 | 9 |
| 6 | Astrid Marie Kolte | 852 | 16 |
| 7 | Yan Zhang | 653 | 28 |
| 8 | Jing Yang | 644 | 35 |
| 9 | Emma S. Lucas | 614 | 10 |
| 10 | Dajin Li | 561 | 16 |

**Table S5. Top 20 keywords related to this** **research field**

| **Rank** | **Keyword** | **Counts** | **Rank** | **Keyword** | **Counts** |
| --- | --- | --- | --- | --- | --- |
| 1 | recurrent miscarriage | 1947 | 11 | implantation | 204 |
| 2 | women | 791 | 12 | spontaneous-abortion | 197 |
| 3 | miscarriage | 709 | 13 | in-vitro fertilization | 153 |
| 4 | expression | 556 | 14 | regulatory t-cells | 139 |
| 5 | pregnancy | 551 | 15 | diagnosis | 137 |
| 6 | risk | 336 | 16 | preeclampsia | 137 |
| 7 | association | 256 | 17 | endometrium | 135 |
| 8 | NK cells | 251 | 18 | invasion | 131 |
| 9 | polymorphism | 228 | 19 | abortion | 124 |
| 10 | infertility | 224 | 20 | trophoblast | 119 |

**Table S6. The top 10 co-cited articles related to this** **research field**

| **Rank** | **Author** | **Title** | **Journal** | **Year** | **Co-citations** |
| --- | --- | --- | --- | --- | --- |
| 1 | Magnus *et al*. | Role of maternal age and pregnancy history in risk of miscarriage: prospective register based study | BMJ | 2019 | 58 |
| 2 | Vento-Tormo *et al*. | Single-cell reconstruction of the early maternal-fetal interface in humans | Nature | 2018 | 51 |
| 3 | Popescu *et al*. | Recurrent pregnancy loss evaluation combined with 24-chromosome microarray of miscarriage tissue provides a probable or definite cause of pregnancy loss in over 90% of patients | Human reproduction | 2018 | 37 |
| 4 | Wang *et al*. | Single-cell Immune Landscape of Human Recurrent Miscarriage | Genomics Proteomics Bioinformatics | 2021 | 36 |
| 5 | Guo *et al*. | Single-cell profiling of the human decidual immune microenvironment in patients with recurrent pregnancy loss | Cell discovery | 2021 | 31 |
| 6 | Lucas *et al*. | Loss of Endometrial Plasticity in Recurrent Pregnancy Loss | Stem cells | 2016 | 29 |
| 7 | Ding *et al*. | The miR-27a-3p/USP25 axis participates in the pathogenesis of recurrent miscarriage by inhibiting trophoblast migration and invasion | Journal of cellular physiology | 2019 | 28 |
| 8 | Lucas *et al*. | Recurrent pregnancy loss is associated with a pro-senescent decidual response during the peri-implantation window | Communications biology | 2020 | 28 |
| 9 | Wang *et al*. | Low chorionic villous succinate accumulation associates with recurrent spontaneous abortion risk | Nature communications | 2021 | 27 |
| 10 | Qian *et al.* | Distinct pattern of Th17/Treg cells in pregnant women with a history of unexplained recurrent spontaneous abortion | Bioscience trends | 2021 | 27 |
